# Exclusive breastfeeding practice and associated factors among mothers of infants age 6 to 12 months in Somali region of Ethiopia

**DOI:** 10.1038/s41598-022-22051-0

**Published:** 2022-11-09

**Authors:** Anguach Shitie, Abebe Tilahun, Lemessa Olijira

**Affiliations:** 1 College of Medicine and Health Sciences, Wollo University, Dessie, Ethiopia; 2 College of Medicine and Health Sciences, Haramaya University, Harar, Ethiopia

**Keywords:** Health care, Public health

## Abstract

In Ethiopia, only 58% of the mothers practice exclusively breast feeding, which is far from recommended; therefore, identifying factors associated with exclusive breast feeding helps to fill this gap. Community-based mixed cross-sectional study was conducted on 532 mothers. Binary logistic regression was performed and Variables with p value ≤ 0.05 in multivariable analysis declared as statistically significant variables. For the qualitative part focused group discussion was performed, and a thematic framework analysis was done. Finally the results were presented with narration. Prevalence of exclusive breastfeeding was 52%. Husband education (AOR = 2.9; 95% CI 1.6, 5), colostrum feeding (AOR = 2.3; 95% CI 1.3, 3.9), antenatal care (AOR = 2.1; 95% CI 1.1, 4.3.), place of delivery (AOR = 2.1, 95% CI 1.2, 3.6), residence (AOR = 0.3; 95% CI 0.2, 0.6), counseling during postnatal care (AOR = 2; 95% CI 1.2, 3.3) were associated with exclusive breastfeeding. As most discussant explained reason for not exclusive breastfeeding were due to different perceptions such as breast milk not sufficient, giving water decrease infantile colic and fear of food refusal. Prevalence of breastfeeding is low. Husband education, residence, colostrum feeding, antenatal care, institutional delivery, counseling during antenatal and postnatal care were significantly associated variables.

## Introduction

Exclusive breastfeeding means that an infant receives only breast milk from his or her mother or a wet nurse or expresses breast milk and no other liquids or solids, not even water, with the exception of oral rehydration solution, drops or syrups consisting of vitamins, mineral supplements or medicines^1^. For almost all infants, breastfeeding remains the easiest, healthiest and least costly method of feeding to meet the needs of infants. However, only 40% of children worldwide exclusively breastfed which is far from the recommendation. In low-income and middle-income countries, only 37% of children exclusively breastfed^2^.

Exclusive breastfeeding is one of the essential actions for infant development and survival. Inadequate breastfeeding severely impacts the health, development and survival of infants, children and mothers^3^. Improving these practices has the potential to save over 820,000 lives per year. Almost half of diarrhea episodes and a third of respiratory infections are caused by inappropriate breastfeeding practices^4^. Prolonged breastfeeding is associated with a 13% reduction in the probability of being overweight and a 35% reduction in the incidence of type 2 diabetes. It is estimated that 20,000 maternal breast cancer deaths could be prevented annually by improving breastfeeding rates. Exclusive breastfeeding also has the potential to prevent 11.6% of under five deaths in developing countries^5^.

Over two-thirds of deaths occurring worldwide during the first year of life of children are often associated with inappropriate feeding practices, especially due to poor exclusive breastfeeding practices. In Asia and Africa, 1.24 million infant deaths occur during the first 6 months of life secondary to poor exclusive breastfeeding practices. It is mainly due to diarrhea and acute respiratory tract infection. Sub-Saharan Africa accounts for 41% of global underfive deaths, mainly due to inadequate breastfeeding practices^6,7^. In Ethiopia, the lack of exclusive breastfeeding is the root cause for 270,000 malnutrition-related deaths of underfive children^8^. It has been reported that the Somali region is among the regions that have the highest rate of infant and underfive mortality caused by diseases related to poor practices of exclusive feeding^9^. Moreover, it also leads to malnutrition, impaired cognitive development, poor school performance and reduced productivity in the future life^10^.

Optimal breast feeding can avert 13% to 15% of underfive deaths. It is recommended that the coverage of the EBF reach 90% to benefit from it^1^. Despite all the recognized advantages and efforts deployed to promote EBF, the practice is still far from the recommended level. In Ethiopia, breast feeding is universal, and 97% of infants are breastfed at some point in time, but only 58% of the mother’s practice exclusively breastfeeding but its prevalence is differ by regions. the prevalence of exclusive breast feeding was 68.8% in Amhara region, Debrebirhan^11^, 82.2% Oromia region, Ambo^12^, 70.5% in southern nation and nationalities, Halaba^13^, and 60.9% in Hawassa^14^, 81.1% in Dubti afar^15^, and 29.3% in Addis Ababa^16^. Various factors have been identified to be associated with the practice of exclusive breastfeeding. factors like age, residence, educational status, antenatal follow up, occupation, are among the commonly listed factors^7^.

The government of Ethiopia has initiated several interventions to improve exclusive breastfeeding practices. National strategy for infant and young child feeding, a national nutrition program I and II, was developed by the government of Ethiopia to promote and improve exclusive breastfeeding practices in the first 6 months in collaboration with different stakeholders^17^.

Pastoralists comprise a significant part of Ethiopia’s population, but the promotion of exclusive breastfeeding and its importance is poorly understood in pastoral communities^18^. Moreover, there is a paucity of information on the practice and determinants of EBF in pastoral communities. Therefore, this study aimed to assess the prevalence of EBF practices and the factors related to EBF practices among mothers of 6-month-old to 1-year-old infants in pastoral communities of Somali region, southeastern Ethiopia, which is vital for health service providers, policy makers and program managers to design intervention strategies that may promote exclusive breastfeeding practices in the study area.

## Methods and materials

### Study area and period

This study was conducted in Chereti district, Afder zone, Somali region, Eastern Ethiopia. The district is approximately 800 km and 1428 km from the capital of the region, Jigjiga and the capital of the country, Addis Ababa, respectively. The total estimated population of the district is approximately 126,228, of which 55,540 are male and 70,688 are female. Of these, 88,652 live in rural areas, while 13,820 live in urban areas. The main economic source of the district is cattle breeding. This study was conducted in the Chereti district of the Somali region, Southeast Ethiopia, from March 11 to April 10, 2019.

### Study design and period

A community-based cross-sectional study mixed with a qualitative study was conducted from March 11 on April 10, 2019.

### Population

#### Source population

All mothers who have 6-month to 12-month infants in Chereti District were the source population.

#### Study population

Mothers who have 6-month to 12-month infants in Chereti District and living in the selected Kebeles.

#### Inclusion criteria

All mothers with 6-month-old to 12-month-old infants and residents of Chereti District for at least 6 months were included in the study.

#### Exclusion criteria

Mothers who were seriously ill and unable to respond at the time of data collection were excluded.

### Sampling procedure

A multistage sampling technique was used to select the study participants. First, the district was stratified into urban and rural kebeles. Then, eight kebeles from 17 rural kebeles and 1 kebele from 2 urban kebeles were selected by the simple random sampling technique. Next, mothers with 6 month- to 1-year-old infants were identified by conducting censuses in the selected kebeles using health extension workers. Based on the number of mothers with 6-month-old to 1-year-old infants, proportional allocation was performed to each kebeles. Study subjects were drawn from the sampling frame by systematic random sampling method. If there were two children in one household from different mothers, only one child was selected randomly and included in the study.

For the qualitative methods, a purposeful sampling method was used for FGD. The participants were selected purposefully based on their role in the community and parity. Focus group discussions were undertaken in a group of grandmothers (the woman who had at least one grandchild), community elders, traditional birth attendants, and mothers (prima para and multipara). Two FGDs each comprised 10 participants. All the FGD participants were selected from kebeles who were not included in the quantitative study group.

During the discussion, a FGD guide with written questions and probes was used. It asked about the definition of exclusive breastfeeding, practices and personal experiences with exclusive breastfeeding, community influences, and colostrum practices, as well as obstacles and solutions for improving exclusive breastfeeding in the community.

### Sample size determination

To determine the sample size, a single population proportion formula was used for the first objective by considering the expected prevalence of EBF of 81.1%, which was taken from a study performed in the Afar region of Ethiopia^19^, with a margin of error of 5% and a 95% confidence interval of ≥ 236. After adding a 10% nonresponse rate, the design effect of the final minimum sample size of 2 was 520.

For the second objective, the StatCalc function of Epi Info version 7 software was used by considering the following assumptions: confidence level of 95%, margin of error of 5%, power of 80% and percent outcome of exposed and percent outcome of unexposed group. Finally, the sample size of occupational status of the mother, mode of delivery, and educational status of the mother became 189, 503, and 532, respectively, after adding a nonresponse rate of 10% and design effect 2. Therefore, the maximum sample size is high for the second objective, which was 532, which is taken as the final sample size.

### Data collection tool, quality control and measurement

A structured questionnaire was used to collect the quantitative data. The questionnaire was adapted from similar published studies with little modification and translated to the Somali language and back translated to English by expertise to maintain consistency of the questionnaire. Before the actual data collection, a pretest was performed on 5% of the sample size out of the selected kebele. Qualitative data were collected by focus group discussion on the factors affecting exclusive breastfeeding practices in the community.

Six diploma nurses were assigned for data collection, and data were collected by face-to-face interviews. One health officer and one BSC nurse were assigned to supervise the activity. Training was given for data collectors and supervised by the principal investigator for two consecutive days. Focus group discussion was performed in the qualitative part of the study. Focus group discussions were facilitated by one BSc nurse who is fluent with the local language and the principal investigator (PI) with the assistance of two diploma nurses (note takers) who are fluent in the Somali language. The notes were written in the Somali language and then translated to English by confirmation with the tape recorder.

### Dependent variables

Exclusive breastfeeding practice.

### Independent variables

Age of the mother, educational status of the mother, educational status of the partner, parity, occupation, marital status, residence, movement in the last 12 months, breast-related health problem, mode of delivery, colostrum feeding, ANC visit, PNC, counseling about exclusive breastfeeding during ANC and PNC, partner support and place delivery were the independent variables.

### Operational definition

#### Exclusive breastfeeding practice

if a mother gives only breast milk for her infant for full 6 months, no other liquids or solids except vitamins, mineral supplements, or medicines until 6 months.

Kebele is the smallest administrative unit of Ethiopia, which consists of at least five hundred families.

### Statistical analysis

Data were checked for completeness, and the response was coded and entered into Epi data version 3.02. Then, the data were exported to the statistical package for social science (SPSS) software version 20 for analysis. Bivariable and multivariable binary logistic regression analyses were used to determine the association of each independent variable with the dependent variable, and variables with a 95% CI and P value < 0.25 during the bivariable analysis were entered into the multivariable analysis to control the effects of confounding variables. Those variables with P values less than or equal to 0.05 in multivariable analysis were considered significant. A multicollinearity test was used to check the linear correlation among the independent variables by using SE. Variables with an SE of > 2 were dropped from the multivariable analysis. The goodness of fit was tested by the Hosmer–Lemeshow statistic (0.93).

Qualitative data were transcribed in English text with the help of a local language speaker (Somaligna). Different ideas in the text were merged in their thematic areas, and a thematic framework analysis was employed using open code software version 4.02. The results were presented in the narratives in triangulation with quantitative data.

### Ethical consideration

All methods were performed in accordance with the relevant guidelines and regulations**.** Before starting the data collection process, Haramaya University College of Health and Medical Science Institutional Health Research Ethics Review Committee (IHRERC) approved ethical clearance. Cooperation letters were obtained from Haramaya University College of Health and Medical Science and were provided to the woreda administration. The Woreda administration had written a supportive letter to the selected kebeles office for their cooperation. Participation was voluntary, and information was collected after obtaining informed, voluntary, written and signed consent from each respondent by assuring confidentiality throughout the data collection period. Participants were told the objective of the study and their right to refuse or answer the questionnaires and were given the right to stop or withdraw at any time of data collection. Confidentiality was maintained.

## Result

### Sociodemographic characteristics of respondents

A total of 529 mothers and infants participated in this study, resulting in a response rate of 99.4%. The mean age of the mother was 25.2 (± 4.7) years. More than two-thirds (197) of the mothers were in the age group of 25–29. All participants were Muslim in religion. Three-fourths of the mothers (75%) were housewives, and less than half of the mothers were not able to read and write (46.7%). Nearly all mothers were married (98.9%), and 44.8% of partners were in primary school and above by educational status. Most of the respondents (84.5%) were living in rural areas (Table 1).

**Table 1:** Socio-demographic characteristics of respondents in chereti district Afder zone southeastern Ethiopia, 2019 (n = 529).

	Category	Frequency	%
Maternal age	16–19	70	13.2
	20–24	170	32.1
	25–29	197	37.2
	≥ 30	92	17.4
Current marital status	Married	526	99.43
	Unmarried	3	1.57
Husband educational status	Unable to read and write	131	24.8
	Able to read and write	161	30.4
	Primary	59	11.2
	Secondary	67	12.7
	College/university	111	21
Maternal educational status	Unable to read and write	247	46.7
	Able to read and write	134	25.3
	primary school	65	12.3
	secondary and above	83	15.7
Occupation of the mother	House wife	405	76.6
	Employed	79	14.9
	Other	45	8.5
Area of resident	Urban	82	15.5
	Rural	447	84.5
Husband advice about breastfeeding	Yes	217	41.02
	No	312	58.0.97
Movement in the last 12 months	Yes	133	25.14
	No	396	74.85
Place of delivery	Health institution	364	68.8
	Home	165	31.19

### Characteristics of children and their mothers

Almost all children (99.4%) were delivered by spontaneous vaginal delivery. Nearly 70% of mothers were assisted by trained health professionals, while the rest were assisted by traditional birth attendants and relatives. More than one-fourth of the mothers (26.84%) had breast-related problems after delivery. Nearly 60% of the mothers had 2–4 children, while one-fifth of the mothers (20.6%) were primiparous, and the rest were grand multiparous. Nearly half of the children in this study were male. The mean (± SD) age of the children was 8.5 (± 1.75) months, and nearly half of the children were in the 6–8 month age group. More than one-fourth (30.8%) of the children were given prelacteal food. Less than half of the children (45.6%) started complementary foods at 6 months and above (Table 2).

**Table 2:** Characteristics of children aged 6 months to 12 months and their mother in Chereti woreda Afder zone Somale region southwestern Ethiopia, 2019.

Variables	Category	Frequency	%
Mode of delivery	Spontaneous vaginal delivery	525	99.24
	Cesarean section	4	0.75
Delivery assistance	Trained health professional	363	68.62
	Other	166	31.37
Breast problem	Yes	142	26.84
	No	387	73.15
Number of children	1	109	20.6
	2–4	308	58.22
	≥ 5	112	21.17
Sex of the child	Male	285	53.87
	Female	244	46.12
Child age in month	6–8	263	49.7
	9–10	173	32.7
	11–12	93	17.6
Giving colostrum	Yes	253	47.8
	No	276	52.2
Prelacteal feeding	Yes	163	30.8
	No	366	69.2
Time additional food starting	Not yet started	55	10.4
	2–3	134	25.7
	4–5	97	18.3
	≥ 6	241	45.6

### Respondent mothers health service utilization

Four hundred thirty-six (82.4%) of the mothers had ANC follow-up during the current child pregnancy. From this, 61.4% (325) of the mothers had 1–3 visits, while 21% had 4 and above visits. Of those mothers with ANC visits, 60.5% were counseled about exclusive breast feeding. A total of 278 (52.6%) patients were receiving postnatal care. Almost all mothers with PNC were counseled about exclusive breastfeeding (Table 3).

**Table 3:** Respondent health service utilization in Chereti district, Afderzone, Somali region, southeastern Ethiopia, 2019 (n = 529).

Variables	Category	Frequency	%
ANC follow-up	Yes	436	82.4
	No	93	17.6
Number of visits	0	93	17.6
	1–3	325	61.4
	4 and above	111	21
Counseling about EBF	Yes	264	49.9
	No	265	50.1
Postnatal care	Yes	278	52.6
	No	251	47.4
Counseling about EBF	Yes	267	96.04
	No	11	3.06

### Magnitude of exclusive breast feeding practice

**Figure 1:**
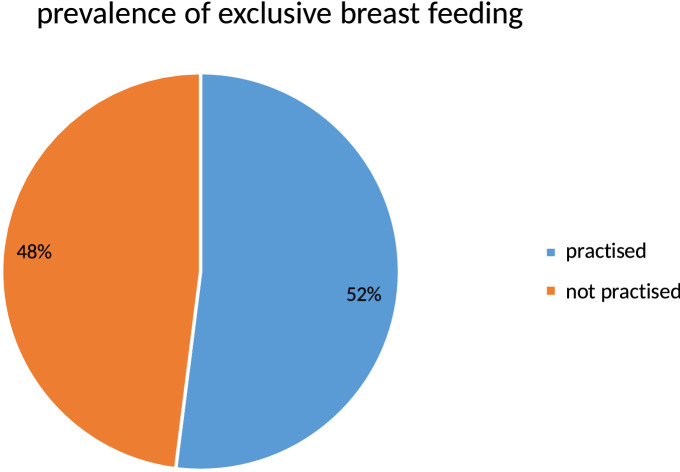
Prevalence of exclusive breast feeding practice among infants aged 6 months to 12 months in Chereti district, Somali region, southwestern Ethiopia, 2019, (n = 529).

In this study, the magnitude of EBF was 52% (95% CI 48%, 57%). Among mothers who did not exclusively breastfeed their infant, the main reason mentioned was that perception of breast milk only being not sufficient for the infant 174 (68.9%) (Fig. 1).

The qualitative findings proved that the majority of the discussants mentioned that breastfeeding is important for the infant, but breast milk alone may not be sufficient for the infant until 6 months. Therefore, the mother should give additional food to her baby; otherwise, the baby be starved, and the baby may refuse to consume food if he/she did not start early.

*An old man from the community leader states that* “*mothers must care for their children by giving breast milk and other additional food after 4 months unless the baby may refuse food after 6 months if she/he didn’t start food early for her baby”*.

*A 27-year old FGD discussant said that “Only breast milk may not be sufficient for the baby until 6 months, so giving additional food and water after 4 months is important for the baby”*.

Some of the discussants mentioned that early initiation and exclusive breast feeding are important for the baby and the mother, so the baby must take only breast milk until 6 months, but after 6 months, the baby should take additional food because after 6 months, only breast milk may not be sufficient.

*An old traditional birth attendant said that “As soon as the child is delivered breast milk giving is must. From birth to 6 months, the child should take only breast milk”*.

Among qualitative findings, both men’s and women’s FGD discussants raised that the reasons for not exclusively breastfeeding were due to different perceptions of the community, such as breast milk only not sufficient, babies thirsty unless they drink water, giving water decrease abdominal cramp or infantile colic, giving only breast milk for the baby affect the mother the mother become wasted, and the baby may refuse food if they do not start early.

*A 34 year old multiparous woman discussant states that “I had never practiced EBF in two of my baby because I believe the breast milk is not sufficient but for the third baby I gave only breast milk for 6 month because the doctor told me to give only breast milk for 6 month“*.

*Another 40 year old discussant said that “I understand that the child should be breastfed for 6 months without mixing but the problem is that he may refuse to take other food after 6 month”.*

### Factors associated with exclusive breast feeding

In the bivariable analysis, factors such as maternal educational status, husband educational status, area of residence, place of delivery, feeding colostrum, having ANC follow up, movement in the previous 12 months, breast problem, Counseling about EBF during ANC and counseling about EBF during PNC were associated with exclusive breast feeding. Mothers whose were able to read and write (AOR = 2.31; 95% CI 1.5, 3.6), attend primary school, (AOR = 2.07 95% CI 1.2, 3.6), secondary school and above (AOR = 3.22; 95% CI 1.9, 5.5), husband’s literacy status able to read and write (AOR = 3.14; 95% CI 1.2, 5.2), primary school (AOR = 7.37 95% CI 3.7, 14.7), secondary school (AOR = 6; 95% CI 3.2, 11.) and college/university(AOR = 4.18; 95% CI 2.4, 0.2), residence **(**AOR = 2.01, 95% CI 0.3, 0.8), Movement in the past 12 month (AOR = 3.8; 95% CI 0.2, 0.4), breast problem (AOR: 3.7; 95% CI 2.5,5.6), colostrum feeding (AOR = 2.5; 95% CI 1.8, 3.5), place of delivery (AOR = 3.0; 95% CI 2.1,4.4), ANC follow up (AOR = 7.2; 95% CI 4.1,12.7) Counseling about EBF during ANC (AOR = 7.6; 95% CI 5.2,11.23), Counseling about EBF during PNC (AOR = 4.6; 95% CI 3.2,6.6) were associated with exclusive breast feeding.

However, in multivariable analysis only, husband literacy status, rural area of residence, feeding colostrum, ANC follow-up, health institutional delivery, counseling about EBF during ANC follow-up and counseling about EBF during postnatal care were significantly associated with exclusive breastfeeding practices. Mothers whose husband’s literacy status was able to read and write, primary school, secondary school and college/university were 2.9 (AOR = 2.9; 95% CI 1.6, 5.5), 4.5 (AOR = 4.5; 95% CI 1.7, 12.1), 5.5 (AOR = 5.5; 95% CI 2.2, 14.2) and 3.6 times (AOR = 3.6; 95% CI 1.5, 8.9) more likely to practice exclusive breastfeeding than those whose husbands were unable to read and write, respectively. Mothers living in urban areas were 67% less likely to practice exclusive breastfeeding than rural mothers (AOR = 0.33; 95% CI 0.2, 0.6). Mothers who were giving colostrum to their infant were 2.3 times more likely to practice exclusive breastfeeding than mothers who did not (AOR = 2.3; 95% CI 1.3, 3.9). Mothers who had antenatal follow-up were 2.1 times more likely to practice exclusive breastfeeding than mothers who did not (AOR = 2.1; 95% CI 1.1,4.3). In addition, mothers who were counseled about exclusive breastfeeding were 3.4 times more likely to practice exclusive breastfeeding than mothers who were not counseled about exclusive breastfeeding (AOR = 3.4; 95% CI 1.9, 6.2). Mothers who gave birth in the health institution were 2.1 times more likely to practice exclusive breastfeeding than those who delivered home (Table 4).

**Table 4:** Factors associated with practice EBF among mothers of children aged between 6 and 12 months during multivariable logistic regression analyses in Cherti district, Afder zone, Somali Region, southeastern Ethiopia, 2019, (n = 529).

Variables	Category	YES	NO	COR 95% CI	AOR 95% CI
		No (%)	No (%)		
Husband educational status	Unable to read and write	35 (26.7)	96 (73.3)	1	1
	Able to read and write	86 (53.4)	75 (46.6)	**3.14 (1.2,5.2)**	2.9 (1.6,5.5)**
	Primary	43 (72.9)	16 (27.1)	**7.37 (3.7,14.7)**	4.5 (1.7,12.1)**
	Secondary	46 (68.7)	21 (31.3)	**6.01 (3.2,11.5)**	5.5 (2.2,14.2)*
	College/university	67 (60.4)	44 (39.6)	**4.18 (2.4,7.2)**	3.6 (1.5,8.9)**
Maternal educational status	Unable to read and write	100 (40.5)	147 (59.5)	1	1
	Able to read and write	82 (62.2)	52 (38.8)	**2.32 (1.5,3.6)**	0.97 (0.5,1.9)
	Primary school	38 (58.5)	27 (41.5)	**2.07 (1.2,3.6)**	0.6 (0.29,1.5)
	Secondary and above	57 (68.7)	26 (31.3)	**3.22 (1.9,5.5)**	1.2 (0.5,1.9)
Area of resident	Urban	55 (67.1)	27 (32.9)	**2.01 (0.3,0.8)**	0.33 (0.2,0.6)*
	Rural	222 (49.7)	225 (50.3)	1	1
Movement in the last 12 months	Yes	38 (28.6)	95 (71.4)	1	1
	No	239 (60.4)	157 (39.6)	**3.8 (0.2,0.4)**	1.1 (0.6,1.9)
Place of delivery	Health institution	221 (60.7)	143 (39.3)	**3.01 (2.1,4.4)**	2.1 (1.2,3.6)**
	Home	56 (33.9)	109 (66.1)	1	1
Breast problem	Yes	43 (29.7)	102 (70.3)	1	1
	No	234 (60.9)	150 (39.1)	**3.7 (2.5,5.6)**	1.7 (0.9,3.2)
Giving colostrum	Yes	162 (64)	91 (36)	**2.5 (1.8,3.5)**	2.3 (1.3,3.9)**
	No	115 (41.7)	161 (58.3)	1	1
ANC	Yes	261 (59.9)	175 (40.1)	**7.2 (4.1,12.7)**	2.1 (1.1,4.3)**
	No	16 (17.2)	77 (82.8)	1	1
Counseling about EBF during ANC	Yes	200 (75.8)	64 (24.2)	**7.6 (5.2,11.23)**	3.4 (1.9,6.2)**
	No	77 (29.1)	188 (70.9)	1	1
Counseling about EBF during PNC	Yes	199 (68.9)	90 (31.1)	**4.6 (3.2,6.6)**	2 (1.2,3.3)*
	No	78 (32.5)	162 (67.5)	1	1

Significant values are in bold.

## Discussion

The prevalence of exclusive breastfeeding in this study was 52% (95% CI 47%, 57%). This result was in line with studies performed in Tamilnadu India (52%)^20^, Kenya wajir (55%)^21^, Indonesia (51.2%)^22^, Mecha district (47.13%)^7^, Motta (50.1%)^23^ and Bahardar (50.03%)^24^.

However, this study was lower than studies done in Ghana (72%)^25^, West Mamprusi District in Northern Ghana (84.3%)^26^, Debrebirhan (68.8%)^11^, Ambo (82.2%)^12^, Halaba (70.5%)^13^, Hawassa (60.9%)^14^, and Dubti afar (81.1%)^15^, and it was also lower than the 2016 EDHS result, which was 58%^9^. Conversely, this study was higher than studies performed in Bangladesh (35.9%)^27^, Saudiarebia (31.1%)^28^ and Addis Ababa (29.3%)^16^. This discrepancy could be due to differences in healthcare coverage and accessibility of health services. The other reason could be the variations in the study period and design used by the researchers. Sociocultural, economic and health care service utilization differences could have also brought these differences nationwide as well as globally.

Among the variables in multivariable analysis, husband literacy status, residence, colostrum feeding, ANC follow-up, counseling about EBF during ANC follow-up, health institution delivery and counseling about EBF during postnatal care were significantly associated with the practice of exclusive breastfeeding.

In this study, mothers whose husbands were literate were more likely to practice exclusive breastfeeding than those whose husbands were unable to read and write. This is supported by similar studies performed in Debre Berhan^11^ and Bahardar^24^. This could be justified because the husband’s literacy status could enable him to understand the importance and benefits of exclusive breastfeeding, and they might have encouraged their partner to exclusively breastfeed their child until 6 months. It should not be forgotten in communities, and support from trained counselors and peers, including other mothers and family members, plays a key role. The support of men, husbands and partners cannot be underestimated^29^. In addition, this is probably because they are better able to comprehend the benefits and consequences within the context of existing customs, traditions, and social and environmental constraints.

Interestingly, urban mothers were less likely to practice exclusive breastfeeding. This is in line with studies performed in Debre Berehan^11^ and Malaysia^30^. This could be explained by the fact that urban mothers have more opportunities for different job opportunities, which limits time to stay with their infants and in turn can compromise EBF practices, or it might be because urban mothers have more access to other infant feeding alternatives than rural mothers.

Moreover, in our analysis, mothers who gave colostrum were more likely to exclusively breastfeed their child than those who did not. This is similar to studies done in Motta^23^. This might be because colostrum increases the infants’ suckling activity, which in turn increases maternal milk secretion to prevent the mother from giving additional food since she fell her baby obtained adequate breast milk. So supporting the mother to feed colostrum for her infant helps to improve practice of exclusive breastfeed till 6 month.

This study also revealed that mothers who had antenatal care follow-up practiced exclusive breast feeding better than mothers who did not. This is similar to studies performed in India Tamul nudu^20^, Northern Ghana^21^, Azezo^31^, Ambo^12^, Halaba^13^ and Dabat^32^. This could be because mothers who had ANC visits may receive different nutritional and other health-related education from health professionals during their follow-up time, which might have a great impact on exclusive breastfeeding. Since the ANC follow-up is a continuing process, mothers could receive much information, and this information may increase the knowledge and attitudinal changes regarding neonatal feeding practice, as well as the nutritional benefits of breast milk for the health of mothers and newborns.

ANC are more likely to practice exclusive breastfeeding than mothers who did not receive counseling during ANC follow-up. This study is supported by studies performed in Tanzania^33^ Azezo^31^, Motta^23^, DebreMarkos^34^, Addis Ababa^16^ and Halaba^13^. This might be explained as Mothers who were counseled during pregnancy prepared themselves psychologically as well economically to exclusively breastfeed the infant. Counseling enhances mothers’ understanding and appreciation of the demands and benefits of EBF. Improving and sustaining breastfeeding support at the household and community levels. Promotion, counseling and education on EBF in the health facility and community was deemed one of the ‘most powerful interventions’ examined to improve breastfeeding, showing a 152% increase in EBF.

In this study, mothers who delivered in health institutions were more likely to practice exclusive breastfeeding than those who delivered at home. This finding is supported by studies done in Azezo^31^ and Hawassa^14^. This could be because mothers who deliver in health institutions might have immediate exposure to health workers, which could help them obtain important messages about child feeding, especially exclusive breastfeeding. The other possible explanation could be that when mothers give birth in health facilities, they can obtain information about a good position and attachment to breastfeeding and support breastfeeding immediately after birth by trained health professionals^29^. This implies that institutional delivery helps to improve the practice of exclusive breastfeeding, so the government should do on institutional delivery to increase the practice of exclusive breastfeeding.

Our analysis showed that mothers who were counseled about EBF during postnatal care were more likely to exclusively breastfeed their children until 6 months than those who did not. This finding is supported by studies performed in Halaba^13^ and Debremarkose^34^*.* This could be due to Support from skilled health care providers through breastfeeding counseling allowing women to make a well-informed decision on whether to breastfeed and to feel confident in her abilities if she decides to breastfeed. The other possible justification could be that breastfeeding counseling also improves mothers’ skills and helps to solve difficulties that may arise during breastfeeding. This could be true in countries that work to ensure infant and young child feeding counseling is available in healthcare facilities and upholding a woman’s right to information and health^35^.

## Limitations of the study

Since the data are self-reported, there may be social desirability bias and recall bias. To mitigate this, we are try to minimize the time which the event is occur the data collector informed the mothers about the issues of confidentiality and used probes for mothers to be open regarding the EBF practices.

## Conclusion

The prevalence of exclusive breastfeeding in this study was lower than the WHO recommendation. Husband literacy status, residing in the rural area, giving colostrum, ANC follow-up, counseling about breastfeeding during ANC and PNC and health institution delivery were significantly associated with exclusive breast feeding. Most of the discussant believed that breast feeding is important for the baby but not sufficient for the baby until 6 months. The most common reason for not practicing exclusive breast feeding is due to different perception of the community, such as breast milk only not sufficient, babies thirsty unless they drink water, giving water decrease abdominal cramp or infantile colic, giving only breast milk for the baby affect the mother the mother become wasted, and the baby may refuse food if they do not start early.

## Recommendation

In this study exclusive breast feeding is lower than the recommendation therefore, further efforts are required to increase the practice of exclusive breastfeeding, by maintain the education of people and strengthen maternal health service utilization, such as antenatal care and institutional delivery, In addition, Ensuring mothers receive consistent support and clear advice from trained and skilled personnel about exclusive breast feeding is very crucial.

## Data Availability

Full data for this research are available through the corresponding author upon request.

## Abbreviations

AIDS: Acquired immune deficiency syndrome
AOR: Adjusted odds ratio
ANC: Antenatal care
CSA: Central Statistical Agency
EBF: Exclusive breastfeeding
EDHS: Ethiopian Demographic Health Survey
HIV: Human immune virus
NGO: Non-governmental organization
SPSS: Statistical package for social science
WHO: World Health Organization
